# CD14 Deficiency Impacts Glucose Homeostasis in Mice through Altered Adrenal Tone

**DOI:** 10.1371/journal.pone.0029688

**Published:** 2012-01-13

**Authors:** James L. Young, Alfonso Mora, Anna Cerny, Michael P. Czech, Bruce Woda, Evelyn A. Kurt-Jones, Robert W. Finberg, Silvia Corvera

**Affiliations:** 1 Program in Molecular Medicine, University of Massachusetts Medical School, Worcester, Massachusetts, United States of America; 2 Interdisciplinary Graduate Program, University of Massachusetts Medical School, Worcester, Massachusetts, United States of America; 3 Department of Medicine, University of Massachusetts Medical School, Worcester, Massachusetts, United States of America; Institut de Pharmacologie et de Biologie Structurale, France

## Abstract

The toll-like receptors comprise one of the most conserved components of the innate immune system, signaling the presence of molecules of microbial origin. It has been proposed that signaling through TLR4, which requires CD14 to recognize bacterial lipopolysaccharide (LPS), may generate low-grade inflammation and thereby affect insulin sensitivity and glucose metabolism. To examine the long-term influence of partial innate immune signaling disruption on glucose homeostasis, we analyzed knockout mice deficient in CD14 backcrossed into the diabetes-prone C57BL6 background at 6 or 12 months of age. *CD14-ko* mice, fed either normal or high-fat diets, displayed significant glucose intolerance compared to wild type controls. They also displayed elevated norepinephrine urinary excretion and increased adrenal medullary volume, as well as an enhanced norepinephrine secretory response to insulin-induced hypoglycemia. These results point out a previously unappreciated crosstalk between innate immune- and sympathoadrenal- systems, which exerts a major long-term effect on glucose homeostasis.

## Introduction

The toll-like receptors (TLR) constitute the initial defense against invading microorganisms, through the recognition of microbial products and activation of innate immune signaling. The recognition of gram-negative bacteria occurs through the specific interaction bacterial lipopolysaccharide (LPS) with TLR4, and the subsequent initiation of a pro-anti-inflammatory response characterized by cytokine induction and secretion. CD14 is a 55 kD membrane-anchored glycoprotein that is absolutely required for the productive interaction of LPS with TLR4 [1], [2]. TLR4 and CD14 are predominantly expressed in immune cells such as macrophages, which initiate the earliest responses to invading microorganisms. However, TLR4 transcripts and protein have been detected in other cell types, including endothelial and neuroendocrine cells, [3], [4], [5], suggesting that cells other than macrophages participate in innate immune signaling. Indeed, cells in the para-ventricular hypothalamus [6], [7], [8], [9], as well as cultured hypothalamic neurons [7] respond very rapidly to LPS administration in-vivo or in-vitro. This responsiveness of the hypothalamic-pituitary and sympathoadrenal axes in response to microbial invasion may play an important role in host defense by rapidly triggering stress response pathways which in turn enhance immune function [10].

The integrated response of the host to LPS can also be affected by physiological modulation of the levels of CD14 and TLR4. For example, TLR4 can be induced in human adipose tissue [11], and its expression in macrophages can be induced several-fold by oxidized LDL [12]. Increased expression of TLR4 and hypersensitivity to LPS is also seen in response to aberrant TGFβ signaling [13]. A role for TLR4 and CD14 in mediating chronic low-level inflammation has also been proposed. Obesity is often accompanied by low-level inflammation, and this could potentially account for the strong association between obesity and insulin resistance [14], [15], [16], [17], [18], [19], [20], [21], [22], [23]. The association between obesity and inflammation could be due to many factors associated with high fat diets, such as changes in gut microbiota, gut permeability, or sensitization to bacterial products [24], [25], [26], [27]. It has also been proposed that saturated free fatty acids, which are elevated in obesity, may generate insulin resistance through mechanisms that require activation of TLR4 and downstream pro-inflammatory signaling pathways [28], [29].

In this context, it would be anticipated that suppression of TLR4 signaling would reduce the deleterious metabolic effects associated with obesity and consumption of high fat diet. However, because physiological systems such as the HPA axis are also responsive to LPS, the suppression of innate immune signaling might influence metabolism in ways unrelated to insulin sensitivity. To better understand the relationships between innate immune signaling and metabolic homeostasis, we studied mice lacking LPS binding capacity through knockout of CD14. These mice fail to mount a pro-inflammatory response to LPS [30], [31], and thus are protected from CD14/TLR4-dependent insulin resistance. In spite of this protection, we find that CD14 knockout mice display significantly impaired glucose tolerance at 6 and 12 months of age. Interestingly, this is accompanied by altered basal adrenal tone and hyper-responsiveness to hypoglycemia-induced sympatho-adrenegic signaling. These results reveal a functional relationship between innate immunity and sympatho-adrenal function, which has a dominant impact on glucose homeostasis.

## Materials and Methods

### Materials

Recombinant human insulin was from Novo Nordisk (Novo Nordisk Inc, Princeton, NJ); Fatty acid free bovine serum albumin (Fraction V), oleic and palmitic acids were from Calbiochem.

### Animals and animal care

All experiments involving vertebrate animals performed in the work shown in this manuscript were described in protocol number 02-20-2009 and were specifically approved by the University of Massachusetts Medical School Institutional Animal Care and Use Committee, (Animal Welfare Assurance Number A3306-01). *CD14-ko* mice have been backcrossed at least 12 generations to the C57BL/6J background. C57Bl/6J mice were obtained from the Jackson Laboratory at 6–11 weeks of age. All mice were housed in ventilated polysulfone cages (Allentown Inc., Allentown, NJ) in a strictly pathogen-free barrier facility maintained on a 12-hour light/12-hour dark cycle. Mice had free access to autoclaved water and food. Obesity was induced by a high fat diet consisting of ∼60% of calories from fat (TD93075; Harlan Teklad, Madison, WI) starting at 11 weeks of age. Prior to 11 weeks, mice were fed the standard pellet diet (LabDiet PicoLab 5053, Purina Mills, St. Louis, MO). Animal weight and food consumption was measured weekly for the duration of the experiment. Animals were fasted for 16–18 hours prior to sacrifice by cervical dislocation followed by bilateral pneumothorax. Harvested tissues were immediately frozen in liquid nitrogen and stored at −80°C or fixed in formalin for immunohistochemical analysis.

### Activity Measurements

Mouse movement was measured in custom-built activity monitors using 880 nm near-infrared light emitting diodes with a scan frequency of 125 Hz and a minimum detection interval of 0.040 seconds. Ambulatory activity was calculated by sequential beam breaks, while stereotypic activity was calculated by multiple breaks of the same beam. Mice were housed individually in their native cages in parallel with controls with an initial 24-hour acclimatization period and were monitored for at least 96 hours.

### Body Fat Analysis

Fat tissue, lean tissue, and free fluid were measured by time domain nuclear magnetic resonance (TD-NMR) using the Bruker LF50 (Bruker Optics Inc, Billerica, MA) which utilizes relative relaxation amplitude and duration to provide tissue contrast.

### Glucose and Insulin Tolerance Tests

Glucose tolerance test was performed with intraperitoneal injection of 10% w/v D-glucose in sterile water (2 g glucose/kg body weight) following a 16–18 hour overnight fast. Whole blood glucose values were measured using Ascencia Breeze (Bayer Healthcare Diabetes Care Division, Tarrytown, NJ) or BD Logic (Becton, Dickinson and Co, Franklin Lakes, NJ) glucose monitors before and after the indicated times post challenge/injection. Insulin tolerance tests were similarly performed with i.p. injection of recombinant human insulin (0.75 U insulin/kg body weight; Novolin R, Novo Nordisk Inc, Princeton, NJ) and blood glucose measurements were done at the indicated times post challenge/injection. Serum insulin was measured using ELISA (Millipore, Billerica, MA). Where indicated, propranolol (2 mg/kg body weight; Sigma, St. Louis, MO) or saline were injected 30 minutes prior to insulin tolerance test protocol initiation.

### Norepinephrine measurement

Norepinephrine was measured according to manufacturer's protocol. In short, plasma noradrenaline was extracted with a cis-diol affinity gel, acylated to N-acylnoradrenaline, converted enzymatically to N-acylnormetanephrine, and quantified in a competitive immunoassay (Alpco Diagnostics, Salem, NH).

### Serum Lipid Analyses

Serum was collected following 9 hour fast via retro-orbital bleeding. Colorimetric analyses were employed to measure total cholesterol (Wako Diagnostics, Richmond, VA), triglyceride (Sigma, St. Louis, MO), and non-esterified fatty acid (Wako Diagnostics) according to manufacturer's protocol.

### Adrenal Gland dissection and size analysis

The ventro-medial adrenal surface was marked with surgical ink to maintain orientation. Adrenal glands were removed under a dissecting microscope with liberal margins to prevent tissue distortion and were immediately fixed in Bouin's solution for 8 hours followed by overnight washing. Tissue was embedded in paraffin and serially sectioned at 8 um intervals. Images (every 10^th^ section starting from the first section) were captured with a Zeiss Axiovert 200 inverted microscope (Thornwood, NY).

### Other methods

C57Bl/6 mice (6–10 weeks of age) were intraperitoneally (i.p.) injected with 4% thioglycollate (Sigma). After four days the animals were euthanized and the peritoneum was flushed with sterile PBS. The peritioneal exudate cells (PECs) were washed and resuspended in DMEM with 10% fetal bovine serum (HyClone), and 0.1% Pen/Strep (Cellgro). Cells were counted using a hemacytometer and plated in a 96-well flat bottom plate at a final concentration of 2×10^5^ cells/200 µL. Palmitic and Oleic acids were conjugated to BSA (fatty acid free, Calbiochem) at a molar ratio of 2∶1. Cells were stimulated with lipids or BSA alone at (1, 5, 10, 20, and 50 µLs) with and without phenol purified LPS (1–100 ng/mL) (Sigma) for 16–20 hrs at 37°C and 5% CO2. Supernatants were collected and an ELISA was performed for mouse IL6 (BD Pharmingen) [32]. Oxygen consumption in response to free fatty acids by cultured C2C12 myotubes was done using a fluorescence-based oxygen consumption assay as described previously [33]. ATP levels were measured using a luciferase-based ATP determination assay (Molecular Probes).

### Statistical Analysis

Statistical analysis employed two-tailed Student's t tests or two-way ANOVA followed by Bonferroni multiple comparisons test. Statistics were performed using Prism Software (Graphpad, San Diego, CA). Results are shown as mean ± SEM unless otherwise stated.

## Results and Discussion

**Figure 1:**
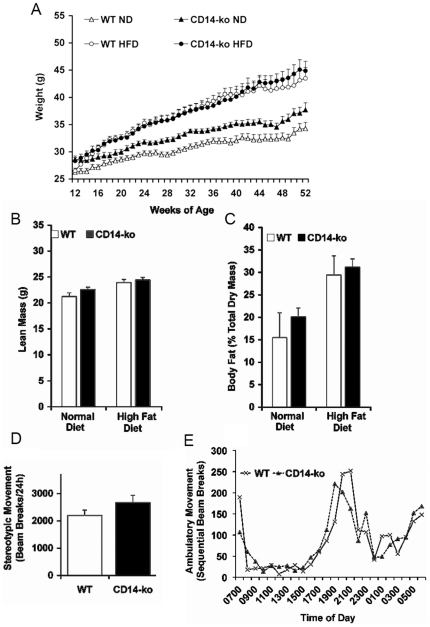
Body weight, adiposity, and activity in wild-type and CD14-ko mice. (A) Growth curves of mice on ND and HFD; NMR analysis of absolute lean mass (B) and fat mass (C) as a percentage of total dry mass. (D) Stereotypic movement determined by consecutive breaks in the same beam path in 26 week old mice on ND. (E) Ambulatory activity as estimated by sequential beam breaks in the x-y axis over a 72-hour period after 24-hour acclimatization in 26 week old mice on ND. Data presented in panels A–D are mean±SEM. (n = 8 mice per group, n = 4 mice for activity measurements). Data in E is a representative experiment which was reproduced 4 times with similar results.

*CD14-ko* mice fed either normal (ND) or high fat (HFD) diet gained weight at a similar rate to the C57Bl/6J control cohort (Fig. 1A), and their weights were not statistically significantly different at the time of metabolic studies. Consistent with this finding, NMR analyses of lean (Fig. 1B) and adipose (Fig. 1C) mass demonstrated no genotype-specific differences between *CD14-ko* mice from controls. Similarly, serum cholesterol, triglyceride, and non-esterified fatty acid (NEFA) in *CD14-ko* mice were not significantly different from wild type mice (Table 1). As total energy expenditure and altered circadian rhythms can contribute to metabolic variation in whole-body knockout mice, all mice studied were individually housed in activity cages [34]. Total spontaneous ambulatory movements and their circadian rhythmicity were indistinguishable from age- matched controls (Fig. 1D,E).

**Table 1:** Lipid Profile and Fasting Insulin of wild-type and CD14-ko mice at 52 weeks of age following 41 weeks of dietary treatment.

Genotype	Diet	Cholesterol (mg/dL)	Triglyceride (mmol/L)	NEFA (mEq/L)	Fasting Insulin (mEq/L)
**WT**	Normal Chow	133.7±12.1	1.61±0.11	0.688±0.16	0.76±0.15
**CD14-KO**	Normal Chow	127.2±8.6	1.62±0.02	0.804±0.05	1.10±0.16
**WT**	High Fat Chow	152.9±14.7	1.52±0.13	0.530±0.08	1.52±0.27^a^
**CD14-KO**	High Fat Chow	140.1±10.9	1.54±0.03	0.536±0.01	2.72±0.55^b^

Values are expressed as mean ± SEM, and significance calculated using ANOVA, n = 6–8.

a , p<0.01 relative to WT ND (normal diet);

b , p<0.01 relative to CD14-ko ND. NEFA, non-esterified fatty acid.

**Figure 2:**
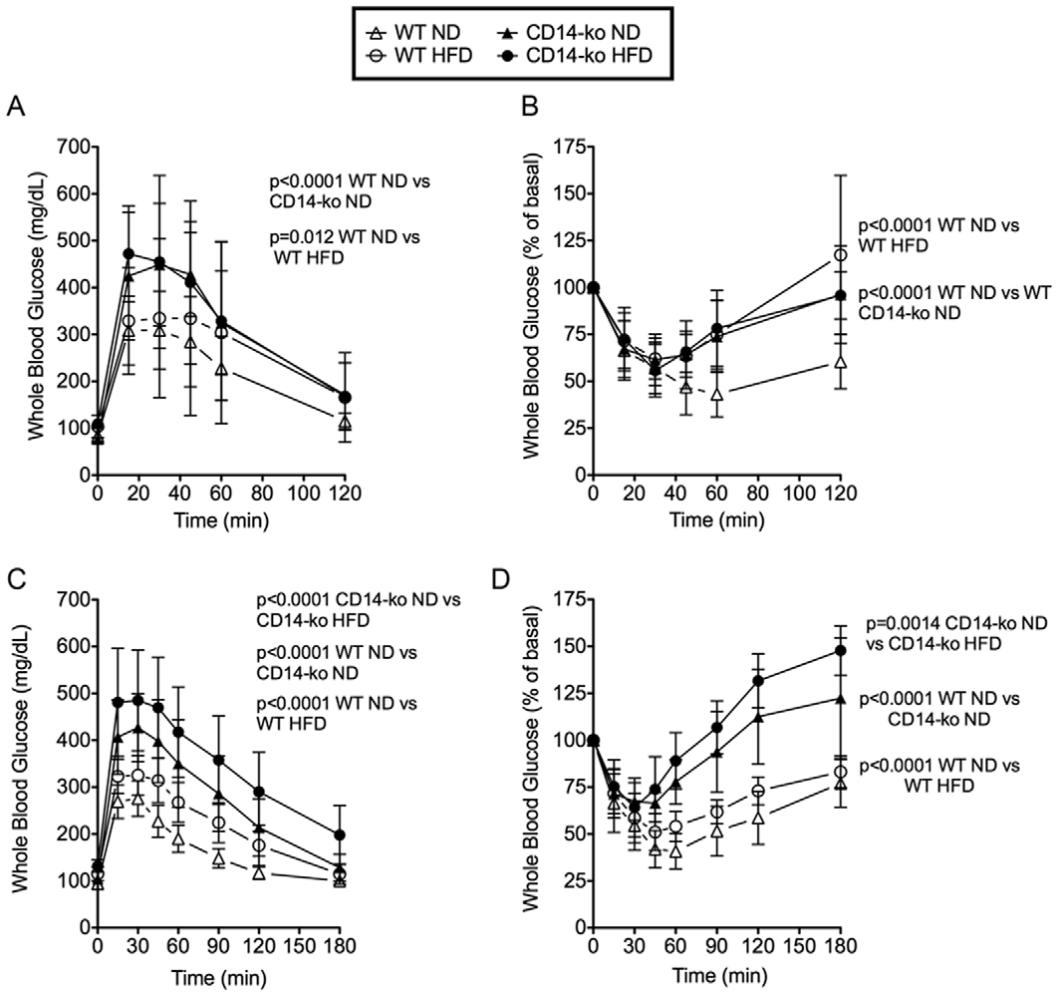
Effects of disruption of the LPS signaling pathway on glucose tolerance. Glucose (A,C) and insulin (B,D) tolerance curves for 26-week (A,B) and 52-week old (C,D) wild-type and *CD14-ko* mice on normal and HFD diets. Statistical differences between groups were determined by 2-way ANOVA, using Bonferroni multiple comparisons test. n = 8 mice per group. The values for basal glucose (mean and SEM in mg/dl, n = 8) for wild-type and *CD14-ko* were: 26 weeks on ND: 80±3 and 77±4; 26 weeks on HFD: 107±8 and 108±4; 52 weeks on ND: 92±1.5 and 104±4; 52 weeks on HFD: 115±5 and 132±5. None of the differences were significant as a function of phenotype.

Despite the lack of genotype-specific changes in overall body composition in *CD14-ko* mice, glucose tolerance tests (GTT) uncovered glucose intolerance. This phenotype was seen in 26-week-old animals (Fig. 2A), and became more pronounced with age, as seen in 52 week-old old mice (Fig. 2C). Glucose tolerance was impaired in response to a HFD in wild-type animals, and was further impaired in *CD14-ko* mice (Fig. 2A). We investigated whether the glucose intolerance seen in these mice could be attributed to insufficient insulin secretion or peripheral insulin resistance. Fasting insulin levels were not significantly different between genotypes (Table 1), although they increased by HFD in both genotypes. *CD14-ko* mice did not have defective insulin secretion, as the time-weighted average insulin values following an i.p. bolus of glucose (2 g/kg body weight) in these mice exceeded that in wildtype controls (3213±344 ng-min/mL in *CD14-ko* vs 1187±82 ng-min/mL in control mice; p<0.005 respectively). Thus, glucose intolerance in *CD14-ko* mice does not appear to be due to impaired insulin secretion.

However, insulin tolerance tests (ITT) did demonstrate genotype-related alterations (Fig. 2B,D). Specifically, while no significant differences were observed during the first 15 min post insulin injection, the rebound phase of the ITT, which reflects the counter regulatory response to hypoglycemia, was significantly enhanced in *CD14-ko* compared to control mice (Fig. 2B). This effect became more pronounced with age (Fig. 2D), and was most pronounced in 52 week-old-mice fed a HFD (Fig. 2D) where, despite similar declines in the first 30 min after insulin injection, blood glucose levels of *CD14-ko* mice were significantly higher than controls after 60 min, and significantly higher than values seen before insulin injection after 90–120 min post-injection (142±8% and 128±4% of initial glucose values at 120 min in 52-week-old WT and *CD14-ko* mice on HFD; P values<0.0001 for *CD14-ko* vs WT, n = 6).

**Figure 3:**
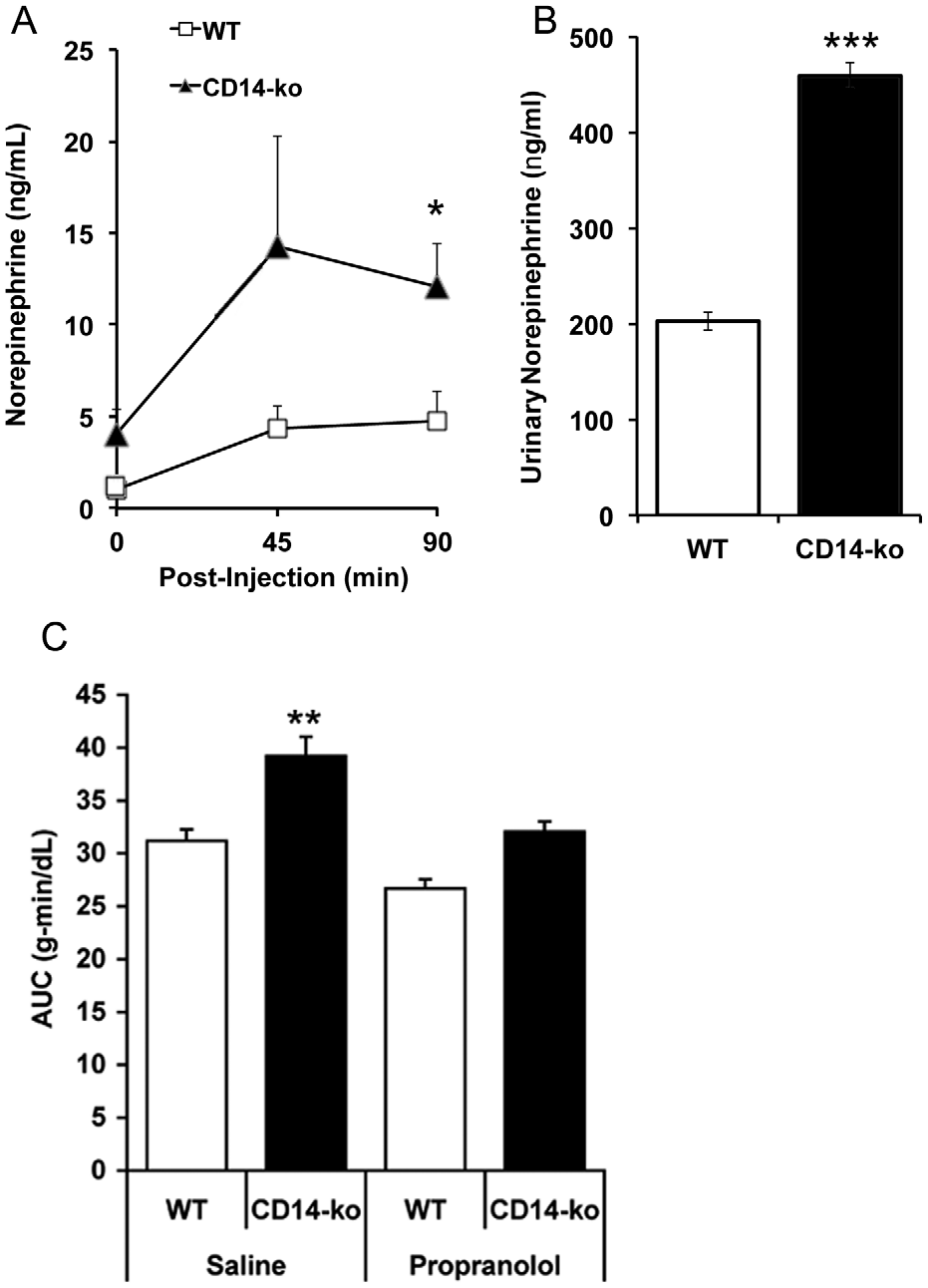
Effects of disruption of the LPS signaling pathway on counter-regulatory response to hypoglycemia. Norepinephrine levels in A) serum of wild type and *CD14-ko* 26 week old mice on ND following injection i.p. with insulin (1–1.5 U/kg body weight) *p<0.05, n = 6; B) urine collected over a 24 h period ***p<0.001, n = 8). C) Area under the curve of the glucose excursions for WT and *CD14-ko* mice following saline or propranolol (2 mg/kg of body weight) injection 30 minutes prior to insulin tolerance tests. **p<0.01 n = 4 for each group. Statistical differences were determined by student t-tests.

Acute counter-regulatory pathways to hypoglycemia rely on adrenal-derived catecholamines to inhibit insulin secretion and peripheral glucose utilization, while promoting hepatic glycogenolysis and stimulating lipolysis [35], [36]. To determine whether adrenal catecholamines may explain the altered rebound phase in the ITT in *CD14-ko* mice, we measured norepinephrine at baseline, at 45 min post-injection to coincide with the inflection in circulating glucose, and at 90 min post-injection when basal glucose levels are re-established. At both 45 and 90 minutes post-injection, norepinephrine levels in *CD14-ko* mice exceeded the values seen in control mice (Fig. 3A). Moreover, increased tonic catecholamine secretion could explain basal glucose intolerance in these mice, as the level of norepinephrine found in urine collected over a 24 hr period was significantly higher in *CD14-ko* mice compared to controls (Fig. 3B). To further test the hypothesis that a hyperactive adrenal response to hypoglycemia contributes to impaired glucose homeostasis in *CD14-ko* mice, mice were treated with the β-adrenergic receptor antagonist propranolol prior to insulin administration. Pre-treatment with propranolol mitigated the excess glucose production in response to insulin-induced hypoglycemia in *CD14-ko* mice (Fig. 3C).

**Figure 4:**
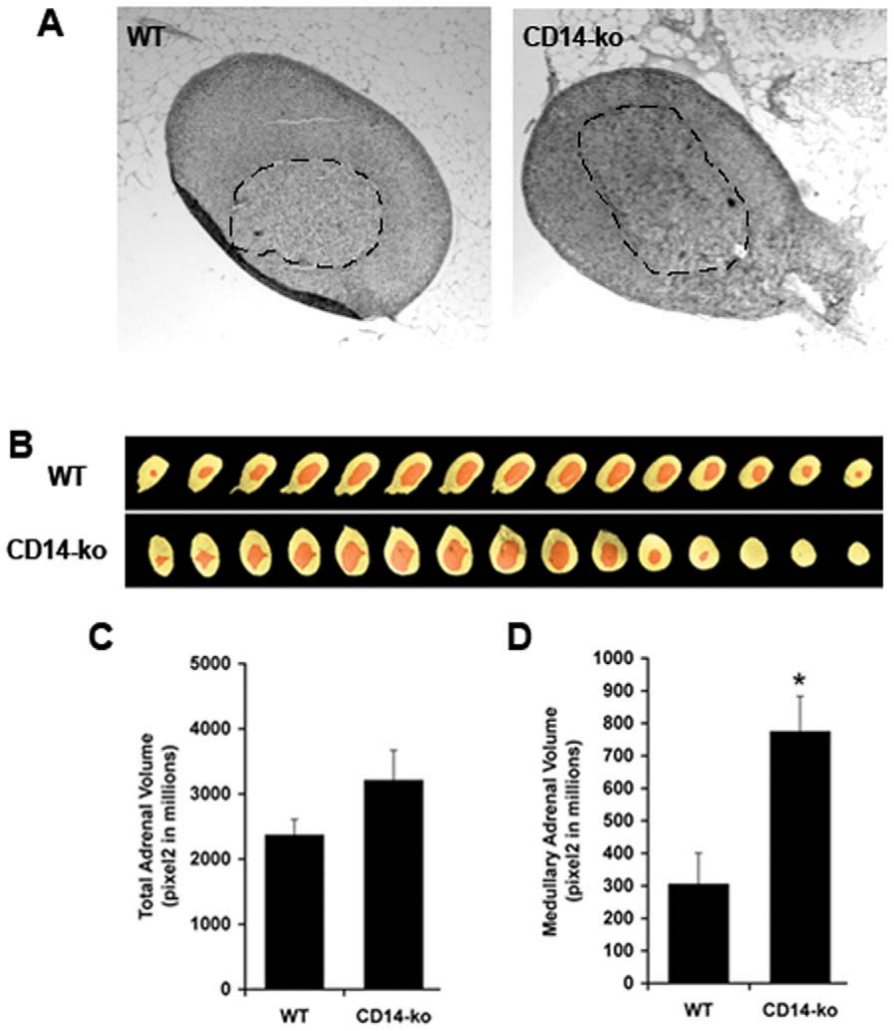
Effects of disruption of the LPS signaling pathway on adrenal gland morphology. A) Adrenal glands were excised, fixed and serially sectioned, and representative sections of a gland from a WT and a CD14-ko mouse are shown. The medulla is outlined by the slashed line. B) Every 10th section was photographed, and in each image the entire gland was pseudocolored yellow, and the medulla was pseudocolored orange. The sum of the areas of all sections were used to estimate total (C) and medullary (D) adrenal volumes in the glands from each genotype. *p<0.05 determined by student t-test, n = 4 adrenal glands per group.

To search for additional evidence for an effect of CD14 depletion on adrenal function that could underlie the observed alteration in sympathoadrenal response to hypoglycemia seen in *CD14-ko* mice, we analyzed the morphology of their adrenal glands. Adrenal glands from 5 mice from each genotype were excised, fixed and serially sectioned at 8 um intervals (Fig. 4A). Every 10th section was photographed (Fig. 4B), and the areas of the whole adrenal and of the medulla in each section were measured. These were summed to obtain a direct estimate of total adrenal and adrenal medullary volume (Fig. 4C,D). This morphometric method was advantageous in that it eliminated variation due to extraneous tissue associated with the gland during dissection. While the total adrenal volume of *CD14-ko* mice, assessed by the sum of area of all sections, was not different from WT mice, (Fig. 4C), the associated adrenal medullary volume was significantly larger (Fig. 4D). Strain-associated variation in adrenal volume has been documented [37]. However, the mice used in these studies were backcrossed for more than 12 generations, making it unlikely that strain-related factors account for the differences observed.

The results presented here suggest that mice lacking a functional LPS signaling pathway become glucose intolerant due to a tonic effect of innate immune signaling to suppress the sympathoadrenal axis, suppressing the production of norepinephrine by the medulla or sympathetic nerve terminals, which produce the majority of norepinephrine in the mouse [38]. The importance of the sympathoadrenal axis on both glucose homeostasis and innate immune signaling is well documented. Insulin sensitivity and glucose uptake are normalized in ob/ob mice by adrenalectomy [39], [40], [41]. In humans, adrenal hyperactivity in Cushing's disease is marked by hyperglycemia and adrenal insufficiency in Addison's disease is marked by hypoglycemia. Thus, enhanced adrenal tone leads to hyperglycemia, through the activation of gluconeogenesis by glucocorticoids, as well as by the sustained counter-regulatory mechanisms mediated by norepinephrine.

Adrenal tone also plays an important role in innate immune signaling. Adrenalectomy results in dramatic hypersensitivity to LPS [42], [43], possibly due at least in part to impairment in catecholamine-mediated protection from LPS-induced hypoglycemia [44], [45]. Also, LPS administration leads to a rapid induction of glucocorticoid production, which is impaired by TLR4 ablation [46], [47], and which is thought to provide a counter regulatory response to mitigate exaggerated inflammatory responses to infection [48], [49]. Because of the strong suppressive effect of catecholamines on LPS-induced inflammatory and metabolic responses, a counter-regulatory input in which CD14/TLR4 signaling might exert a tonic suppression of adrenergic tone might be necessary to maintain adequate innate immune responsiveness. This may be the basis of the phenotype reported in this manuscript, where absence of CD14, which is expected to impair TLR4 signaling, causes enhanced medullary adrenal output.

Several studies have reported that deletion of TLR4 ameliorates insulin resistance produced by a high fat diet [28], [29], [50], [51], [52], [53], [54], raising the question of why in our studies *CD14-ko* mice display enhanced insulin resistance. The amelioration of insulin resistance in TLR4-deficient mice has been interpreted to reflect a direct activation of TLR4 by saturated fatty acids present in the diet. However, the lack of direct interaction between the extracellular domain of TLR4 and saturated fatty acids suggests alternative possibilities [55], [56]. One such possibility may be that some high fat diets may enhance the levels or activity of natural TLR4 ligands such as LPS, for example through changes in gut permeability, microbiota composition, or metabolic endotoxemia [25], [26], [57]. Thus, high-fat diets could produce insulin resistance through at least two concurrent mechanisms; first, by increasing LPS levels or sensitivity to LPS, and second, through direct impairment of insulin signaling pathways by excess free fatty acids [58], [59], which may be enhanced by tonic TLR4 activity. Depending on the relative predominance of these mechanisms, ablation of TLR4 signaling would result in greater or lesser amelioration of HFD induced insulin resistance. This model could explain the results presented here where the absence of CD14 in extensively backcrossed C57Bl6 mice raised and fed in strict pathogen-free conditions does not ameliorate HFD-induced insulin resistance.

**Figure 5:**
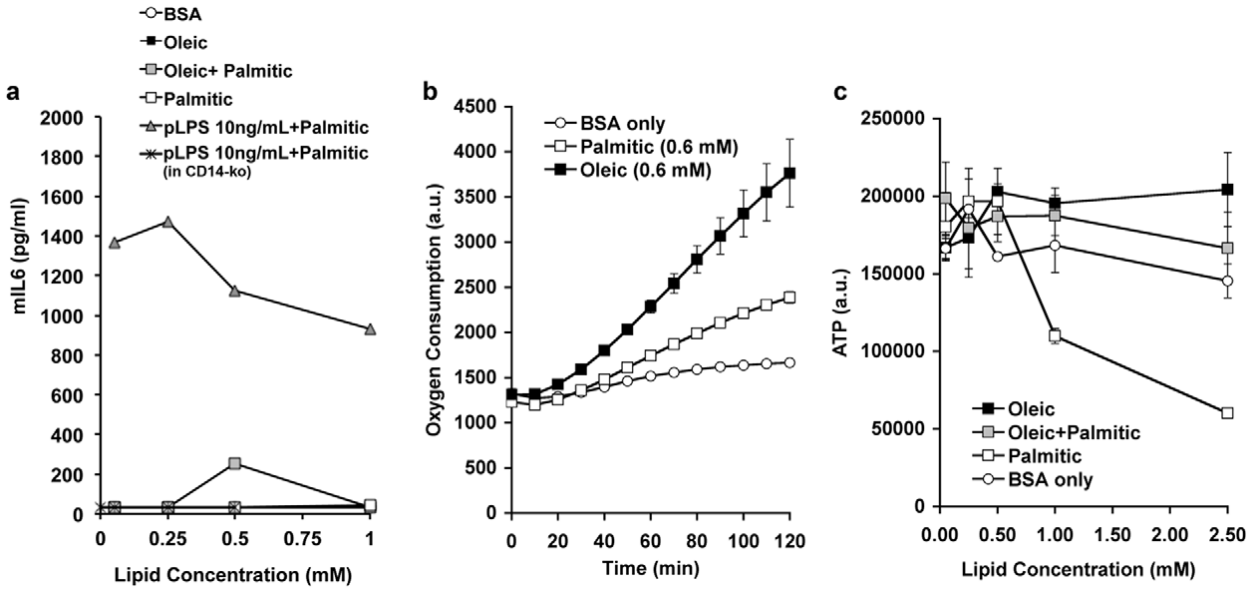
Effect of free fatty acids on peritoneal macrophages. A) IL6 secretion from peritoneal macrophages obtained from wild type or CD14-ko mice incubated in the absence or presence of the indicated concentrations of BSA alone, BSA-conjugated fatty acids, or LPS in the presence of increasing concentrations of palmitic acid. B) Oxygen consumption by C2C12 cells incubated in the absence or presence of the indicated concentration of BSA-conjugated fatty acids, C) ATP levels in peritoneal macrophages obtained from wild type mice incubated in the absence or presence of the indicated concentrations of BSA alone or BSA-conjugated fatty acids. Shown are the means of duplicates from representative experiments which were repeated a minimum of three times with similar results.

This model would also predict that the effects of fatty acids to induce inflammatory responsiveness in macrophages would be negligible compared to those induced by LPS. To test this directly, we compared the induction of IL6 protein secretion by primary macrophages in response to fatty acids and LPS. As expected, secretion of IL6 by peritoneal macrophages was potently stimulated by LPS (Fig. 5A), and this effect was absent in macrophages obtained from *CD14-ko* mice. However, significant IL6 secretion in response to palmitic acid, oleic acid or both could not be detected in this assay, indicating that the magnitude of the effect is very small compared to that of LPS. These fatty acids were functional, as they induced a potent increase in oxygen consumption when added to cultured myocytes, stemming from their entry into the beta-oxidation pathway (Fig. 5B). Also, at high concentrations, palmitic acid had toxic effects on macrophages, reflected by a gradual decline in ATP levels over time (Fig. 5C). These results confirm that LPS activation of TLR4 is much more potent that its possible activation by fatty acids, consistent to that seen by others [55], [56]. Under our experimental conditions the impairment of glucose homeostasis due to lack of CD14/TLR4 signaling overrides the small beneficial effects that might ensue from the mitigation of non-LPS induced pro-inflammatory signaling.

In human populations, high TLR expression is associated with greater inflammatory responses [60]. Conversely, TLR4 polymorphisms (Asp299Gly) that confer decreased responsiveness to LPS [61], [62] increase susceptibility to infection [63], [64]. These polymorphisms are strongly associated with a decreased susceptibility to atherosclerosis [65], consistent with the possibility that the decrease in chronic inflammation due to hyporesponsive alleles suppresses atherogenesis. Interestingly, these same polymorphisms are associated with increased insulin levels, decreased insulin sensitivity and family history of diabetes [66]. In large cross sectional studies the association of these TLR4 alleles with decreased insulin sensitivity persisted when corrected for body fat [67]. Whether these polymorphisms in humans are associated with altered adrenal tone is not known, but they are consistent with our findings of impaired glucose tolerance in animals deficient in innate immune inflammatory signaling. Future studies designed to address the potential role of the LPS signaling pathway in modulating adrenal tone in humans will be required to establish the significance of the results presented here to human metabolic disease.
